# The crystal structure of 3-chloro-2-(4-methyl­phenyl)-2*H*-pyrazolo­[3,4-*b*]quinoline

**DOI:** 10.1107/S205698901500818X

**Published:** 2015-04-30

**Authors:** Haliwana B. V. Sowmya, Tholappanavara H. Suresha Kumara, Jerry P. Jasinski, Sean P. Millikan, Hemmige S. Yathirajan, Christopher Glidewell

**Affiliations:** a PG Department of Chemistry, Jain University, 52 Bellary Road, Hebbal, Bangalore 560 024, India; b University B.D.T. College of Engineering, (a Constituent College of VTU, Belgaum), Davanagere 577 004, India; c Department of Chemistry, Keene State College, 229 Main Street, Keene, NH 03435-2001, USA; d Department of Studies in Chemistry, University of Mysore, Manasagangotri, Mysore 570 006, India; e School of Chemistry, University of St Andrews, St Andrews, Fife KY16 9ST, Scotland

**Keywords:** crystal structure, pyrazolo­quinoline, hydrogen bonding, π–π stacking inter­actions

## Abstract

In the mol­ecule of 3-chloro-2-(4-methyl­phen­yl)-2*H*-pyrazolo­[3,4-*b*]quinoline, C_17_H_12_ClN_3_, (I), the dihedral angle between the planes of the pyrazole ring and the methyl­ated phenyl ring is 54.25 (9)°. The bond distances in the fused tricyclic system provide evidence for 10-π delocalization in the pyrazolo­pyridine portion of the mol­ecule, with diene character in the fused carbocyclic ring. In the crystal, mol­ecules of (I) are linked by two independent C—H⋯N hydrogen bonds, forming sheets containing centrosymmetric *R*_2_^2^(16) and *R*_6_^4^(28) rings, and these sheets are all linked together by π–π stacking inter­actions with a ring-centroid separation of 3.5891 (9) Å.

## Abstract 2

The crystal structure and supra­molecular features of 3-chloro-2-(4-methyl­phen­yl)-2*H*-pyrazolo­[3,4-*b*]quinoline are reported.

## Chemical context   

Quinoline exhibits anti­malarial, anti-bacterial, anti­fungal, anthelmintic, cardiotonic, anti­convulsant, anti-inflammatory and analgesic activity (Marella *et al.*, 2013 ▸). Quinoline and its fused heterocyclic derivatives constitute an important class of compounds for new drug development (Kumar *et al.*, 2009 ▸), and the medicinal applications of pyrazolo­[3,4-*b*]quinolines have been summarized, along with an efficient synthetic method (Afghan *et al.*, 2009 ▸). Recently, we have reported the synthesis of a number of novel pyrazolo­[3,4-*b*]quinoline derivatives, including that of the title compound (I), and mol­ecular docking studies of their binding affinity to the active sites of human telomerase (Sowmya *et al.*, 2014 ▸). In a continuation of that study, we now report the crystal and mol­ecular structure of one such example, the title compound 3-chloro-2-*p*-tolyl-2*H*-pyrazolo­[3,4-*b*]quinoline, (I).
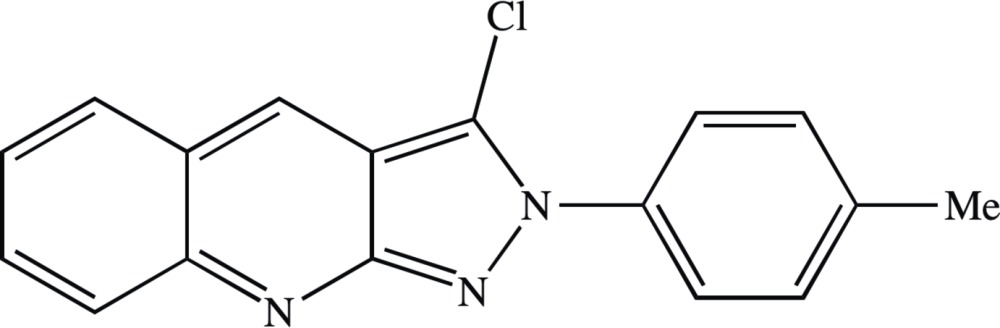


## Structural commentary   

**Figure 1:**
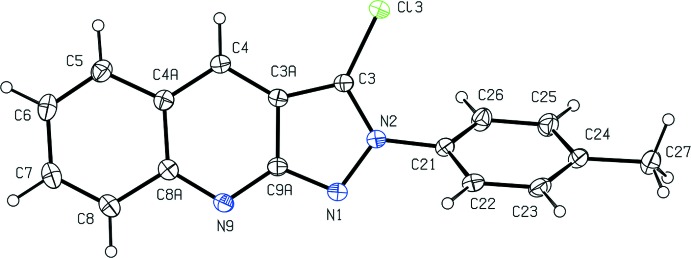
The mol­ecular structure of compound (I), showing the atom-labelling scheme. Displacement ellipsoids are drawn at the 30% probability level.

Within the mol­ecule of compound (I) (Fig. 1 ▸), the pendent phenyl group is twisted out of the plane of the fused heterocyclic ring system, as indicated by the relevant torsional angles (Table 1 ▸): the dihedral angle between the mean planes of the pyrazole and the methyl­ated phenyl rings is 54.25 (9)°. The mol­ecules of (I) exhibit no inter­nal symmetry and thus they are conformationally chiral: however, the centrosymmetric space group accommodates equal numbers of both of the conformational enanti­omers. The non-planar character of the mol­ecular skeleton may be plausibly ascribed to the combined effects of the intra­molecular non-bonded repulsion between the Cl substituent and the nearest H atom of the methyl­ated phenyl ring, and of the direction-specific inter­molecular inter­actions, in particular the hydrogen bonds.

The bond distances in compound (I) (Table 1 ▸) show some inter­esting features. Within the pyrazole ring, the bond distances N1—C9*A* and N2—C3 (Fig. 1 ▸) are identical within experimental uncertainty, although these two bonds are formally double and single bonds, respectively. In the fused carbocyclic ring, the bonds C5—C6 and C7—C8 are much shorted than any other C—C bonds in the mol­ecule. However, in the central pyridine ring, within each of the pairs of corresponding bonds C3*A*—C4 and C4—C4*A*, C8*A*—N9 and N9—C9*A*, and C3*A*—C9*A* and C4*A*—C8*A*, the two distances are very similar. These observations taken together are fully consistent with a 10-π delocalized system in the pyrazolo­pyridine portion of the mol­ecule, comparable to those found in naphthalene and azulene (Glidewell & Lloyd, 1984 ▸), while the fused carbocyclic ring has more the character of an isolated diene (*cf.* Glidewell & Lloyd, 1986 ▸).

## Supra­molecular features   

**Figure 2:**
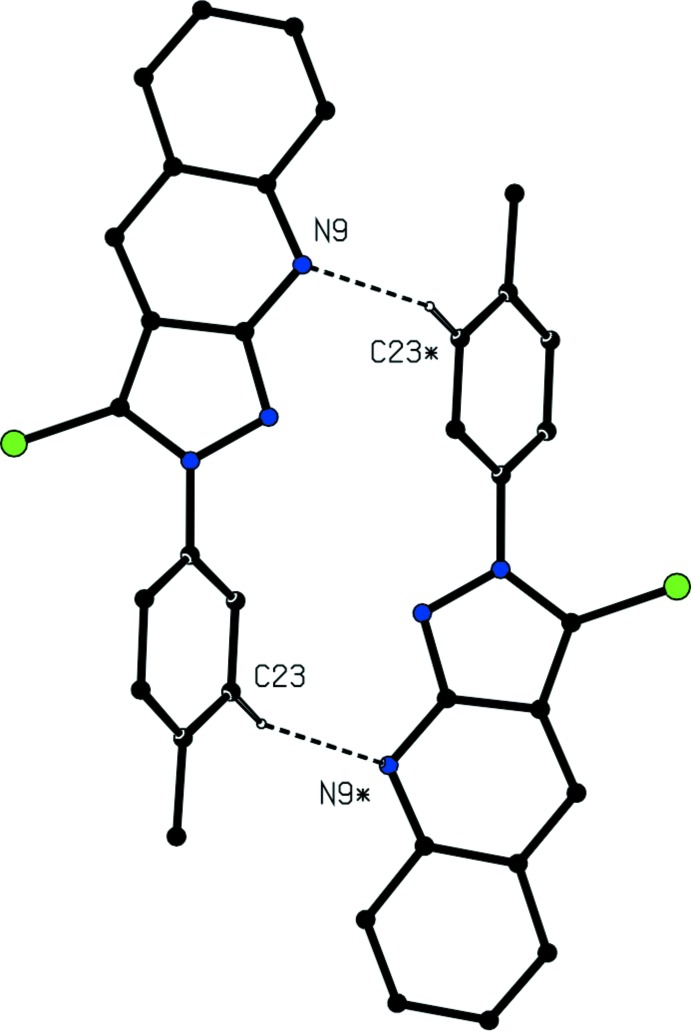
Part of the crystal structure of compound (I) showing the formation of a centrosymmetric hydrogen-bonded dimer. For the sake of clarity, the unit-cell outline and H atoms not involved in the motif shown have been omitted. Atoms marked with an asterisk (*) are at the symmetry position (−*x*, 1 − *y*, 1 − *z*).

**Figure 3:**
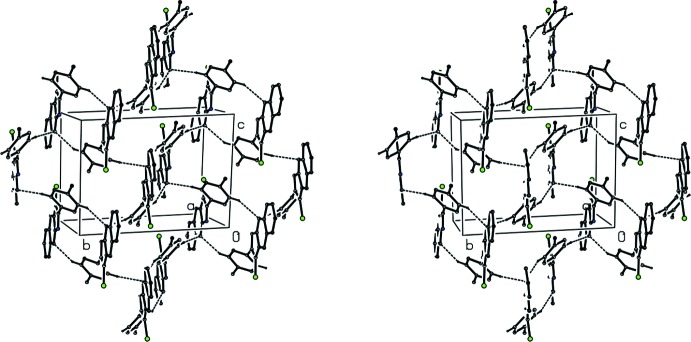
A stereoview of part of the crystal structure of compound (I) showing the formation of a hydrogen-bonded sheet lying parallel to (100) and containing alternating 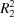
(16) and 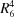
(28) rings. For the sake of clarity, H atoms not involved in the motifs shown have been omitted.

The supra­molecular assembly in compound (I) is determined by two independent C—H⋯N hydrogen bonds (Table 2 ▸) and a π–π stacking inter­action, which together link the mol­ecules into a three-dimensional framework structure. The formation of this framework is readily analysed in terms of three simpler sub-structures (Ferguson *et al.*, 1998*a* ▸,*b* ▸; Gregson *et al.*, 2000 ▸). In the simplest sub-structure, the C—H⋯N hydrogen bond having atom C23 as the donor links an inversion-related pair of mol­ecules, forming a cyclic centrosymmetric dimer characterized by an 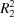
(16) (Bernstein *et al.*, 1995 ▸) motif (Fig. 2 ▸), and this dimeric unit can be regarded as the basic building block in the supra­molecular assembly. The second C—H⋯N hydrogen bond, having atom C26 as the donor, directly links the reference dimer, which is centred at (0, ½, ½) to four symmetry-related dimers centred at (0, 0, 0), (0 1, 0), (0, 0, 1) and (0, 1, 1), thereby leading to the formation of a hydrogen-bonded sheet lying parallel to (100), in which centrosymmetric 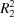
(16) rings alternate with 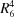
(28) rings (Fig. 3 ▸).

**Figure 4:**
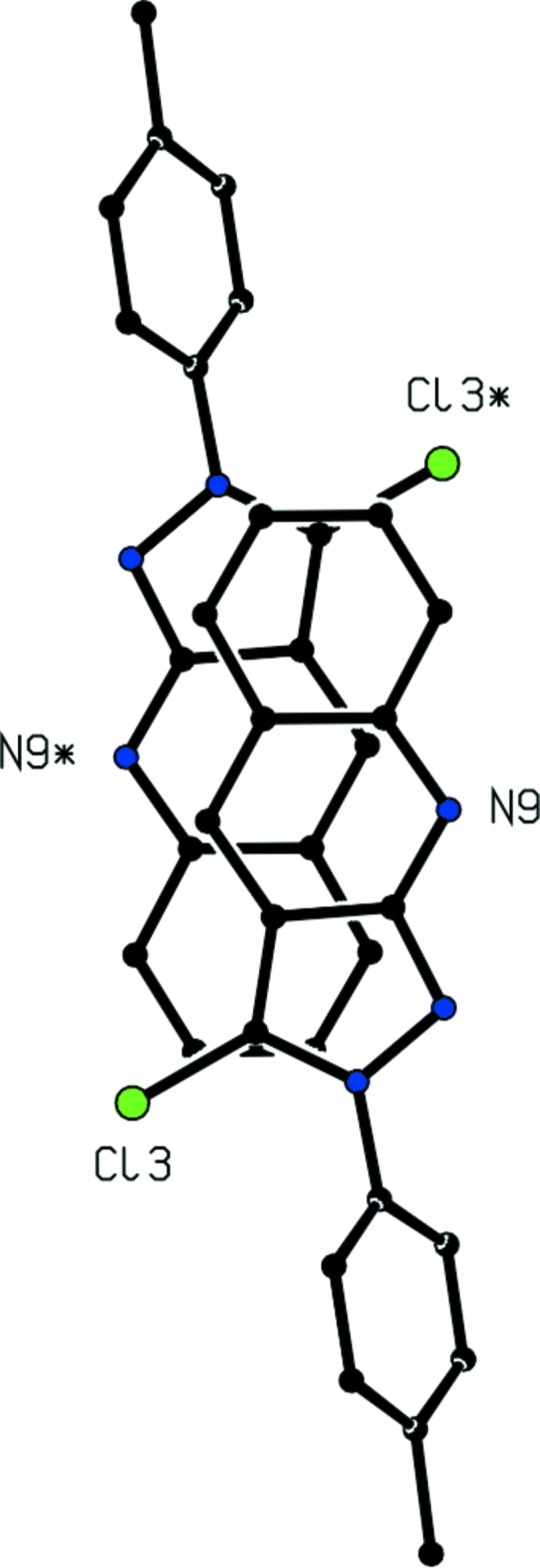
Part of the crystal structure of compound (I) showing the overlap of an inversion-related pair of mol­ecules. For the sake of clarity, the unit-cell outline and all of the H atoms have been omitted. The mol­ecules are viewed normal to the planes of the fused heterocyclic ring system and atoms marked with an asterisk (*) are at the symmetry position (1 − *x*, 1 − *y*, 1 − *z*).

Only one hydrogen-bonded sheet passes through each unit cell, but the sheets are linked by the π–π stacking inter­action which is associated with the extensive overlap between the tricyclic ring systems of inversion-related pairs of mol­ecules in adjacent sheets (Fig. 4 ▸). The pyridine rings of the mol­ecules at (*x*, *y*, *z*) and (1 − *x*, 1 − *y*, 1 − *z*), which lie in adjacent sheets, are strictly parallel with an inter­planar spacing of 3.3819 (6) Å. The ring-centroid separation is 3.5891 (9) Å, corresponding to a ring-centroid offset of *ca* 1.202 Å (Fig. 4 ▸). The effect of this inter­action is to link all of the hydrogen-bonded sheets into a single three-dimensional array.

Despite the large number of aromatic C—H bonds in the mol­ecule of compound (I), the only short C—H⋯π contact involves one of the C—H bonds of the methyl group. Not only are such bonds of low acidity but, perhaps more important, such a methyl group will be undergoing very rapid rotation about the adjacent C—C bond. When a group having local *C*_3_ symmetry, such as a methyl group, is directly bonded to another group having local *C*_2_ symmetry, such as a phenyl group, as in (I), the rotational barrier about the bond between them is very low, generally of the order of J mol^−1^ rather than the usual kJ mol^−1^ (Naylor & Wilson, 1957 ▸; Tannenbaum *et al.*, 1956 ▸). Moreover, it has been shown that simple hydro­carbyl substituents undergo rapid rotation about C—C bonds in the solid state, even at reduced temperatures (Riddell & Rogerson, 1996 ▸, 1997 ▸). Therefore, while such a C—H⋯π inter­molecular inter­action may not be regarded as structurally significant, we report it here for completeness (Table 2 ▸).

## Database survey   

Structural information on un-reduced pyrazolo­[3,4-*b*]quinolines carrying a substituent at the N2 position but not at N1, is sparse. In a series of pyrazolo­[3,4-*b*]quinolin-5-ones, each carrying a substituent at N2, the central heterocyclic ring is in reduced form, carrying H atoms at positions 4 and 8 (Cannon *et al.*, 2001*a* ▸,*b* ▸,*c* ▸,*d* ▸). By contrast, in a series of less highly reduced pyrazolo­[3,4-*b*]quinolin-5-ones which each carry a substituent at N1 but not at N2, the central fused ring is fully aromatic (Mera *et al.*, 2005 ▸; Cruz *et al.*, 2006 ▸; Portilla *et al.*, 2007 ▸). Similarly, in a series of benzo[*f*]pyrazolo­[3,4-*b*]quinolines, in each of which there is a substituent at position 1, but not at position 2, the pyridine ring is fully aromatic (Portilla, Quiroga *et al.*, 2005 ▸; Portilla, Serrano *et al.*, 2005 ▸; Portilla *et al.*, 2008 ▸).

## Synthesis and crystallization   

A sample of the title compound was prepared using the recently published procedure (Sowmya *et al.*, 2014 ▸). Crystals suitable for single-crystal X-ray diffraction were obtained by slow evaporation, at ambient temperature and in the presence of air, of a solution in hexa­ne–ethyl acetate (19:1, *v*/*v*).

## Refinement   

Crystal data, data collection and structure refinement details are summarized in Table 3 ▸. All H atoms were located in difference maps, and then treated as riding atoms in geometrically idealized positions with C—H distances 0.95 Å (aromatic) or 0.98 Å (meth­yl) and with *U*_iso_(H) = *kU*_eq_(C), where *k* = 1.5 for the methyl group, which was permitted to rotate but not to tilt, and 1.2 for all other H atoms.

## Supplementary Material

Crystal structure: contains datablock(s) global, I. DOI: 10.1107/S205698901500818X/sj5454sup1.cif

Structure factors: contains datablock(s) I. DOI: 10.1107/S205698901500818X/sj5454Isup2.hkl

Supporting information file. DOI: 10.1107/S205698901500818X/sj5454Isup3.cml

CCDC reference: 1012361

Additional supporting information:  crystallographic information; 3D view; checkCIF report

## Figures and Tables

**Table 1:** Selected geometric parameters (, )

N1N2	1.3644(18)	C7C8	1.358(2)
N2C3	1.346(2)	C8C8*A*	1.432(2)
C3C3*A*	1.398(2)	C8*A*N9	1.342(2)
C3*A*C4	1.388(2)	N9C9*A*	1.346(2)
C4C4*A*	1.394(2)	C9*A*N1	1.349(2)
C4*A*C5	1.429(2)	C3*A*C9*A*	1.430(2)
C5C6	1.357(3)	C4*A*C8*A*	1.446(2)
C6C7	1.419(3)	C3Cl3	1.6993(16)
			
N1N2C21C22	53.7(2)	C3N2C21C22	125.08(17)
N1N2C21C26	126.42(16)	C3N2C21C26	54.8(2)

**Table 2:** Hydrogen-bond geometry (, ) *Cg*1 represents the centroid of the C21C26 ring.

*D*H*A*	*D*H	H*A*	*D* *A*	*D*H*A*
C23H23N9^i^	0.95	2.50	3.393(2)	157
C26H26N9^ii^	0.95	2.50	3.449(2)	174
C27H27*A* *Cg*1^iii^	0.98	2.84	3.653(2)	140

Symmetry codes: (i) 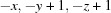; (ii) 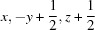; (iii) 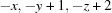.

**Table 3:** Experimental details

Crystal data	
Chemical formula	C_17_H_12_ClN_3_
*M*_r_	293.75
Crystal system, space group	Monoclinic, *P*2_1_/*c*
Temperature (K)	173
*a*, *b*, *c* ()	10.2194(4), 13.4661(5), 10.4600(4)
()	102.780(4)
*V* (^3^)	1403.80(10)
*Z*	4
Radiation type	Cu *K*
(mm^1^)	2.36
Crystal size (mm)	0.42 0.28 0.12

Data collection	
Diffractometer	Agilent Eos Gemini
Absorption correction	Multi-scan (*CrysAlis RED*; Agilent, 2012 ▸)
*T*_min_, *T*_max_	0.554, 0.753
No. of measured, independent and observed [*I* > 2(*I*)] reflections	8443, 2738, 2479
*R*_int_	0.032
(sin /)_max_ (^1^)	0.619

Refinement	
*R*[*F* ^2^ > 2(*F* ^2^)], *wR*(*F* ^2^), *S*	0.040, 0.114, 1.05
No. of reflections	2738
No. of parameters	191
H-atom treatment	H-atom parameters constrained
_max_, _min_ (e ^3^)	0.36, 0.22

Computer programs: *CrysAlis PRO* and *CrysAlis RED* (Agilent, 2012 ▸), *SHELXS97* (Sheldrick, 2008 ▸), *SHELXL2014* (Sheldrick, 2015 ▸) and *PLATON* (Spek, 2009 ▸).

## supplementary crystallographic information

### Crystal data

**Table:**

C_17_H_12_ClN_3_	*F*(000) = 608
*M**_r_* = 293.75	*D*_x_ = 1.390 Mg m^−^^3^
Monoclinic, *P*2_1_/*c*	Cu *K*α radiation, λ = 1.54184 Å
*a* = 10.2194 (4) Å	Cell parameters from 2738 reflections
*b* = 13.4661 (5) Å	θ = 4.4–72.6°
*c* = 10.4600 (4) Å	µ = 2.36 mm^−^^1^
β = 102.780 (4)°	*T* = 173 K
*V* = 1403.80 (10) Å^3^	Block, yellow
*Z* = 4	0.42 × 0.28 × 0.12 mm

### Data collection

**Table:**

Agilent Eos Gemini diffractometer	2479 reflections with *I* > 2σ(*I*)
Radiation source: Enhance (Cu) X-ray Source	*R*_int_ = 0.032
ω scans	θ_max_ = 72.6°, θ_min_ = 4.4°
Absorption correction: multi-scan (*CrysAlis RED*; Agilent, 2012)	*h* = −12→12
*T*_min_ = 0.554, *T*_max_ = 0.753	*k* = −12→16
8443 measured reflections	*l* = −12→11
2738 independent reflections	

### Refinement

**Table:**

Refinement on *F*^2^	0 restraints
Least-squares matrix: full	Hydrogen site location: inferred from neighbouring sites
*R*[*F*^2^ > 2σ(*F*^2^)] = 0.040	H-atom parameters constrained
*wR*(*F*^2^) = 0.114	*w* = 1/[σ^2^(*F*_o_^2^) + (0.0702*P*)^2^ + 0.2814*P*] where *P* = (*F*_o_^2^ + 2*F*_c_^2^)/3
*S* = 1.05	(Δ/σ)_max_ < 0.001
2738 reflections	Δρ_max_ = 0.36 e Å^−^^3^
191 parameters	Δρ_min_ = −0.21 e Å^−^^3^

### Special details

**Table:**

Geometry. All e.s.d.'s (except the e.s.d. in the dihedral angle between two l.s. planes) are estimated using the full covariance matrix. The cell e.s.d.'s are taken into account individually in the estimation of e.s.d.'s in distances, angles and torsion angles; correlations between e.s.d.'s in cell parameters are only used when they are defined by crystal symmetry. An approximate (isotropic) treatment of cell e.s.d.'s is used for estimating e.s.d.'s involving l.s. planes.

### Fractional atomic coordinates and isotropic or equivalent isotropic displacement parameters (Å^2^)

**Table:**

	*x*	*y*	*z*	*U*_iso_*/*U*_eq_	
N1	0.18699 (13)	0.38247 (10)	0.54035 (13)	0.0290 (3)	
N2	0.21652 (13)	0.38697 (10)	0.67397 (13)	0.0269 (3)	
C3	0.34907 (16)	0.38742 (11)	0.72750 (16)	0.0271 (3)	
Cl3	0.41552 (4)	0.39736 (3)	0.89093 (3)	0.03435 (15)	
C3A	0.41587 (16)	0.38246 (11)	0.62459 (15)	0.0257 (3)	
C4	0.54815 (16)	0.37944 (11)	0.61183 (15)	0.0280 (3)	
H4	0.6210	0.3825	0.6860	0.034*	
C4A	0.56909 (16)	0.37163 (11)	0.48495 (16)	0.0287 (3)	
C5	0.70051 (17)	0.36552 (13)	0.45927 (18)	0.0356 (4)	
H5	0.7767	0.3680	0.5302	0.043*	
C6	0.71795 (19)	0.35618 (14)	0.33496 (19)	0.0401 (4)	
H6	0.8060	0.3516	0.3197	0.048*	
C7	0.60533 (19)	0.35325 (13)	0.22768 (18)	0.0390 (4)	
H7	0.6191	0.3472	0.1411	0.047*	
C8	0.47823 (19)	0.35900 (12)	0.24644 (16)	0.0352 (4)	
H8	0.4045	0.3569	0.1730	0.042*	
C8A	0.45426 (17)	0.36816 (11)	0.37577 (15)	0.0282 (3)	
N9	0.32609 (13)	0.37210 (10)	0.38697 (13)	0.0283 (3)	
C9A	0.30883 (16)	0.37944 (11)	0.51055 (15)	0.0264 (3)	
C21	0.10959 (16)	0.39242 (12)	0.74232 (16)	0.0283 (3)	
C22	0.01295 (16)	0.46631 (12)	0.70896 (16)	0.0318 (3)	
H22	0.0147	0.5109	0.6390	0.038*	
C23	−0.08594 (16)	0.47332 (13)	0.78014 (17)	0.0356 (4)	
H23	−0.1520	0.5239	0.7588	0.043*	
C24	−0.09102 (16)	0.40803 (14)	0.88226 (17)	0.0353 (4)	
C25	0.00467 (17)	0.33313 (14)	0.90984 (18)	0.0393 (4)	
H25	0.0016	0.2870	0.9779	0.047*	
C26	0.10438 (17)	0.32453 (13)	0.83996 (17)	0.0360 (4)	
H26	0.1685	0.2725	0.8590	0.043*	
C27	−0.19679 (19)	0.41851 (18)	0.9614 (2)	0.0476 (5)	
H27A	−0.1545	0.4387	1.0510	0.071*	
H27B	−0.2424	0.3547	0.9636	0.071*	
H27C	−0.2623	0.4689	0.9212	0.071*	

### Atomic displacement parameters (Å^2^)

**Table:**

	*U*^11^	*U*^22^	*U*^33^	*U*^12^	*U*^13^	*U*^23^
N1	0.0265 (7)	0.0343 (7)	0.0250 (7)	−0.0008 (5)	0.0028 (5)	−0.0009 (5)
N2	0.0244 (7)	0.0303 (7)	0.0251 (7)	0.0004 (5)	0.0035 (5)	−0.0002 (5)
C3	0.0251 (8)	0.0298 (8)	0.0251 (8)	0.0001 (6)	0.0026 (6)	−0.0006 (6)
Cl3	0.0305 (2)	0.0482 (3)	0.0228 (2)	0.00019 (15)	0.00256 (16)	−0.00138 (14)
C3A	0.0272 (8)	0.0244 (7)	0.0243 (8)	−0.0007 (5)	0.0034 (6)	−0.0001 (6)
C4	0.0261 (8)	0.0288 (7)	0.0278 (8)	−0.0013 (6)	0.0033 (6)	−0.0009 (6)
C4A	0.0298 (8)	0.0243 (7)	0.0322 (8)	−0.0025 (6)	0.0074 (7)	−0.0008 (6)
C5	0.0304 (9)	0.0361 (9)	0.0411 (9)	−0.0026 (7)	0.0093 (7)	−0.0028 (7)
C6	0.0366 (9)	0.0391 (9)	0.0503 (11)	−0.0026 (7)	0.0220 (8)	−0.0036 (8)
C7	0.0500 (11)	0.0363 (9)	0.0359 (9)	−0.0041 (8)	0.0204 (8)	−0.0028 (7)
C8	0.0445 (10)	0.0335 (9)	0.0288 (8)	−0.0032 (7)	0.0109 (7)	−0.0009 (7)
C8A	0.0336 (8)	0.0226 (7)	0.0287 (8)	−0.0014 (6)	0.0078 (6)	0.0003 (6)
N9	0.0306 (7)	0.0293 (7)	0.0243 (7)	−0.0020 (5)	0.0042 (5)	−0.0003 (5)
C9A	0.0270 (8)	0.0246 (7)	0.0267 (8)	−0.0008 (6)	0.0038 (6)	−0.0002 (6)
C21	0.0230 (7)	0.0330 (8)	0.0283 (8)	0.0005 (6)	0.0045 (6)	−0.0020 (6)
C22	0.0266 (8)	0.0352 (8)	0.0305 (8)	0.0015 (6)	−0.0003 (6)	0.0016 (7)
C23	0.0230 (8)	0.0413 (9)	0.0397 (9)	0.0047 (6)	0.0005 (7)	−0.0042 (7)
C24	0.0236 (8)	0.0463 (10)	0.0355 (9)	−0.0018 (6)	0.0053 (7)	−0.0075 (7)
C25	0.0320 (9)	0.0486 (10)	0.0388 (9)	0.0009 (7)	0.0111 (7)	0.0095 (8)
C26	0.0300 (8)	0.0378 (9)	0.0418 (10)	0.0067 (7)	0.0114 (7)	0.0084 (7)
C27	0.0302 (9)	0.0671 (13)	0.0483 (11)	−0.0017 (8)	0.0145 (8)	−0.0121 (10)

### Geometric parameters (Å, º)

**Table:**

N1—N2	1.3644 (18)	C7—H7	0.9500
N2—C3	1.346 (2)	C8—H8	0.9500
C3—C3A	1.398 (2)	N2—C21	1.434 (2)
C3A—C4	1.388 (2)	C3—Cl3	1.6993 (16)
C4—C4A	1.394 (2)	C21—C26	1.380 (2)
C4A—C5	1.429 (2)	C21—C22	1.391 (2)
C5—C6	1.357 (3)	C22—C23	1.385 (2)
C6—C7	1.419 (3)	C22—H22	0.9500
C7—C8	1.358 (2)	C23—C24	1.393 (3)
C8—C8A	1.432 (2)	C23—H23	0.9500
C8A—N9	1.342 (2)	C24—C25	1.390 (3)
N9—C9A	1.346 (2)	C24—C27	1.507 (2)
C9A—N1	1.349 (2)	C25—C26	1.384 (2)
C3A—C9A	1.430 (2)	C25—H25	0.9500
C4A—C8A	1.446 (2)	C26—H26	0.9500
C4—H4	0.9500	C27—H27A	0.9800
C5—H5	0.9500	C27—H27B	0.9800
C6—H6	0.9500	C27—H27C	0.9800
			
C9A—N1—N2	103.40 (12)	C8—C8A—C4A	118.05 (15)
C3—N2—N1	113.60 (13)	C8A—N9—C9A	115.12 (14)
C3—N2—C21	126.87 (14)	N9—C9A—N1	123.20 (14)
N1—N2—C21	119.52 (12)	N9—C9A—C3A	124.38 (14)
N2—C3—C3A	107.30 (14)	N1—C9A—C3A	112.41 (14)
N2—C3—Cl3	124.06 (12)	C26—C21—C22	121.19 (15)
C3A—C3—Cl3	128.59 (13)	C26—C21—N2	119.56 (14)
C4—C3A—C3	136.66 (15)	C22—C21—N2	119.25 (14)
C4—C3A—C9A	120.04 (14)	C23—C22—C21	118.42 (16)
C3—C3A—C9A	103.30 (14)	C23—C22—H22	120.8
C3A—C4—C4A	116.84 (15)	C21—C22—H22	120.8
C3A—C4—H4	121.6	C22—C23—C24	121.64 (15)
C4A—C4—H4	121.6	C22—C23—H23	119.2
C4—C4A—C5	122.13 (16)	C24—C23—H23	119.2
C4—C4A—C8A	119.06 (15)	C25—C24—C23	118.24 (15)
C5—C4A—C8A	118.81 (15)	C25—C24—C27	120.74 (17)
C6—C5—C4A	120.91 (17)	C23—C24—C27	121.02 (17)
C6—C5—H5	119.5	C26—C25—C24	121.16 (16)
C4A—C5—H5	119.5	C26—C25—H25	119.4
C5—C6—C7	120.32 (17)	C24—C25—H25	119.4
C5—C6—H6	119.8	C21—C26—C25	119.26 (16)
C7—C6—H6	119.8	C21—C26—H26	120.4
C8—C7—C6	121.22 (16)	C25—C26—H26	120.4
C8—C7—H7	119.4	C24—C27—H27A	109.5
C6—C7—H7	119.4	C24—C27—H27B	109.5
C7—C8—C8A	120.68 (17)	H27A—C27—H27B	109.5
C7—C8—H8	119.7	C24—C27—H27C	109.5
C8A—C8—H8	119.7	H27A—C27—H27C	109.5
N9—C8A—C8	117.40 (15)	H27B—C27—H27C	109.5
N9—C8A—C4A	124.55 (14)		
			
C9A—N1—N2—C3	0.34 (16)	C8—C8A—N9—C9A	179.20 (14)
C9A—N1—N2—C21	179.24 (13)	C4A—C8A—N9—C9A	−0.2 (2)
N1—N2—C3—C3A	−0.29 (17)	C8A—N9—C9A—N1	−178.78 (14)
C21—N2—C3—C3A	−179.08 (14)	C8A—N9—C9A—C3A	−0.4 (2)
N1—N2—C3—Cl3	177.38 (11)	N2—N1—C9A—N9	178.30 (14)
C21—N2—C3—Cl3	−1.4 (2)	N2—N1—C9A—C3A	−0.27 (16)
N2—C3—C3A—C4	−179.33 (17)	C4—C3A—C9A—N9	1.1 (2)
Cl3—C3—C3A—C4	3.1 (3)	C3—C3A—C9A—N9	−178.44 (14)
N2—C3—C3A—C9A	0.10 (16)	C4—C3A—C9A—N1	179.66 (14)
Cl3—C3—C3A—C9A	−177.43 (12)	C3—C3A—C9A—N1	0.11 (17)
C3—C3A—C4—C4A	178.21 (17)	N1—N2—C21—C22	−53.7 (2)
C9A—C3A—C4—C4A	−1.1 (2)	N1—N2—C21—C26	126.42 (16)
C3A—C4—C4A—C5	−178.51 (14)	C3—N2—C21—C22	125.08 (17)
C3A—C4—C4A—C8A	0.6 (2)	C3—N2—C21—C26	−54.8 (2)
C4—C4A—C5—C6	178.81 (16)	C26—C21—C22—C23	2.9 (2)
C8A—C4A—C5—C6	−0.3 (2)	N2—C21—C22—C23	−177.01 (14)
C4A—C5—C6—C7	0.6 (3)	C21—C22—C23—C24	−0.6 (3)
C5—C6—C7—C8	−0.4 (3)	C22—C23—C24—C25	−1.5 (3)
C6—C7—C8—C8A	0.0 (3)	C22—C23—C24—C27	178.15 (16)
C7—C8—C8A—N9	−179.12 (15)	C23—C24—C25—C26	1.4 (3)
C7—C8—C8A—C4A	0.3 (2)	C27—C24—C25—C26	−178.25 (17)
C4—C4A—C8A—N9	0.1 (2)	C22—C21—C26—C25	−3.0 (3)
C5—C4A—C8A—N9	179.25 (14)	N2—C21—C26—C25	176.89 (16)
C4—C4A—C8A—C8	−179.31 (14)	C24—C25—C26—C21	0.8 (3)
C5—C4A—C8A—C8	−0.2 (2)		

### Hydrogen-bond geometry (Å, º)

Cg1 represents the centroid of the C21–C26 ring.

**Table:**

*D*—H···*A*	*D*—H	H···*A*	*D*···*A*	*D*—H···*A*
C23—H23···N9^i^	0.95	2.50	3.393 (2)	157
C26—H26···N9^ii^	0.95	2.50	3.449 (2)	174
C27—H27*A*···*Cg*1^iii^	0.98	2.84	3.653 (2)	140

Symmetry codes: (i) −*x*, −*y*+1, −*z*+1; (ii) *x*, −*y*+1/2, *z*+1/2; (iii) −*x*, −*y*+1, −*z*+2.
